# Supplementary material for: Statistical and machine learning models for predicting university dropout and scholarship impact
Source: PLoS One. 2025 Jun 25;20(6):e0325047. doi: 10.1371/journal.pone.0325047 (PMC12193850; doi:10.1371/journal.pone.0325047)
Supplement: S1 Table — (DOCX) [file pone.0325047.s001.docx]

| **Class of variable** | **Variable** | **Type** | **Median** | **Mean** ± **SD** |
| --- | --- | --- | --- | --- |
| Demographic data | Age at enrollment (x5) | Numeric/discrete (years) | 20.00 | 23.47 ± 7.93 |
| Macroeconomic data | Inflation rate (x12) | Numeric/continuous (%) | 1.40 | 1.26 ± 1.39 |
| Academic data at enrollment | Application order (x1)  Previous qualification (x2)  Previous qualification (grade) (x3)  Admission grade (x4) | Numeric/ordinal  Numeric/discrete  Numeric/continuous (0-200 scale)  Numeric/continuous (0-200 scale) | 1.00  2.00  133.10  126.10 | 1.79 ± 1.36  2.05 ± 0.61  132.55 ± 13.05  126.68 ± 14.31 |
| Academic data at the end of 1^st^ semester | Curricular units 1^st^ sem (without evaluations) (x6) | Numeric/discrete (0-20 scale) | 0.00 | 0.12 ± 0.62 |
| Academic data at the end of 2^nd^ semester | Curricular units 2^nd^ sem (credited) (x7)  Curricular units 2^nd^ sem (enrolled) (x8)  Curricular units 2^nd^ sem (evaluations) (x9)  Curricular units 2^nd^ sem (grade) (x10)  Curricular units 2^nd^ sem (without evaluations) (x11) | Numeric/discrete (0-20 scale)  Numeric/discrete (0-20 scale)  Numeric/discrete (0-20 scale)  Numeric/continuous (0-20 scale)  Numeric/discrete (0-20 scale) | 0.00  6.00  8.00  12.33  0.00 | 0.55 ± 2.02  6.30 ± 2.27  7.70 ± 3.92  10.03 ± 5.49  0.13 ± 0.68 |

**Table 4. Summary of continuous variables**
