# Supplementary material for: Statistical and machine learning models for predicting university dropout and scholarship impact
Source: PLoS One. 2025 Jun 25;20(6):e0325047. doi: 10.1371/journal.pone.0325047 (PMC12193850; doi:10.1371/journal.pone.0325047)
Supplement: S2 Table — (DOCX) [file pone.0325047.s002.docx]

**Table 5. Summary of categorical variables**

| **Class of variable** | **Variable** | **Type** | **Level** | **Count** |
| --- | --- | --- | --- | --- |
| Demographic data | Marital status (x13) | Numeric/nominal | 1 ─ Single  2 ─ Married  3 ─ Widower  4 ─ Divorced  5 ─ Facto union  6 ─ Legally separated | 2,983  315  2  73  22  5 |
|  | International (x24) | Numeric/binary | 1 ─ Yes  0 ─ No | 80  3,320 |
|  | Displaced (x19) | Numeric/binary | 1 ─Yes  0 ─No | 1,877  1,523 |
|  | Gender (x22) | Numeric/binary | 1 ─Male  0 ─Female | 1,141  2,259 |
| Socioeconomic data | Mother’s occupation (x17) | Numeric/nominal | 0 — Student  1 — Representatives of the Government, Directors and Executive Managers  2 — Specialists in Intellectual and Scientific Activities  3 — Intermediate Level Technicians and Professions  4 — Administrative staff  5 — Personal Services, Security and Safety Workers  6 — Farmers and Skilled Workers in Agriculture, Fisheries and Forestry  7 — Skilled Workers in Industry, Construction and Craftsmen  8 — Installation and Machine Operators and Assembly Workers  9 — Unskilled Workers  10 — Armed Forces Profession  90 — Other | 132  80  215  251  611  417  65  219  28  1,266  2  114 |
|  | Father’s occupation (x18) | Numeric/nominal | 0 — Student  1 — Representatives of the Government, Directors and Executive Managers  2 — Specialists in Intellectual and Scientific Activities  3 — Intermediate Level Technicians and Professions  4 — Administrative staff  5 — Personal Services, Security and Safety Workers  6 — Farmers and Skilled Workers in Agriculture, Fisheries and Forestry  7 — Skilled Workers in Industry, Construction and Craftsmen  8 — Installation and Machine Operators and Assembly Workers  9 — Unskilled Workers  10 — Armed Forces Profession  90 — Other | 115  101  133  294  286  399  165  530  245  830  193  109 |
|  | Debtor (x20) | Numeric/binary | 1 ─ Yes  0 ─ No | 373  3,027 |
|  | Tuition Fees Up-to-date (x21) | Numeric/binary | 1 ─ Yes  0 ─ No | 2,952  448 |
|  | Scholarship holder (x23) | Numeric/binary | 1 ─ Yes  0 ─ No | 930  2,470 |
| Academic data at enrollment | Application mode (x14) | Numeric/nominal | 1 ─ 1^st^ phase – general contingent  7 ─ Holders of other higher courses  17 ─ 2^nd^ phase – general contingent  18 ─ 3^rd^ phase – general contingent  39 ─ Over 23 years old  43 ─ Change of course  44 ─ Technological specialization diploma holders  60 ─ Other | 1,386  126  705  108  650  218  8  199 |
|  | Course (x15) | Numeric/nominal | 1 ─ Buisness  2 ─ Health and human services  3 ─ Professional studies and fine art  4 ─ Sciences | 684  1,197  969  550 |
|  | Daytime/evening attendance (x16) | Numeric/binary | 1 ─ Yes  0 ─ No | 3,015  385 |
| Target | Target (x25) | Numeric/binary | 1 ─ Dropout  0 ─ Graduated | 1,313  2,087 |
