# Supplementary material for: Statistical and machine learning models for predicting university dropout and scholarship impact
Source: PLoS One. 2025 Jun 25;20(6):e0325047. doi: 10.1371/journal.pone.0325047 (PMC12193850; doi:10.1371/journal.pone.0325047)
Supplement: Attachment — Submitted filename: responses_to_reviewers.pdf [file pone.0325047.s006.pdf]

Responses to Referee Comments

We the authors are very thankful to the two reviewers and the Editor for their suggestions and comments, which we believe greatly help improve the presentation of this paper.

Referee 1's Comments

- *The study presents the results of original research. The work presents original findings that advance the study of university dropout prediction. Its results align with the prevailing lines of academic inquiry, contributing further to the development of predictive models for university drop out.*

  Response: Thanks.

- *Results reported have not been published elsewhere. The study employs methodologies that have been applied and validated in other research. While the results have not been previously published, it should be noted that the authors need to improve and deepen their review of the literature. Some relevant studies, published in indexed journals on the subject, have been overlooked and could strengthen their research and results.*

  1. Silva, E. C., Freitas, S., Soares Ramos, C., Muniz De Menezes, A. E., & Rodrigues De Araujo, L. K. S. (2023). A systematic review of the factors that impact the prediction of retention and dropout in higher education.

  2. Ortiz-Lozano, J. M., Aparicio-Chueca, P., Triadó-Ivern, X. M., & Arroyo-Barrigüete, J. L. (2024). Early dropout predictors in social sciences and management degree students. Studies in Higher Education, 49(8), 1303-1316.

  3. Ortiz-Lozano, J. M., Rua-Vieites, A., Bilbao-Calabuig, P., & Casadesús-Fa, M. (2020). University student retention: Best time and data to identify undergraduate students at risk of dropout. Innovations in education and teaching international.

  4. Huynh-Cam, T. T., Chen, L. S., & Lu, T. C. (2024). Early prediction models and crucial factor extraction for first-year undergraduate student dropouts. Journal of Applied Research in Higher Education.

  Response: We cited these studies from Line 28 to Line 65. The new paragraph is written as below: "Ortiz-Lozano et al. [8] focused on investigating the optimal time and types of data needed to identify students who are at risk of dropping out. The study analyzed demographic and academic information from 935 first-year students at an engineering school in Spain with classification tree, which had good interpretability due to its decision tree graph. The prediction accuracy was 76%. The key finding was that academic performance data served as a strong predictor of student withdrawal. Their research supported conclusions in previous studies about supporting the need for an early first-year intervention to improve retention. Ortiz-Lozano et al. [9] analyzed academic data from 3,583 first-year students on the Business Administration (BA) degree at the University of Barcelona and identified that the percentage of failed or unattended subjects in the first semester plays a significant role in predicting dropout. Several machine learning algorithms were applied, namely, neural networks, random forest, and logistic regression. All models exhibited good prediction accuracy and sensitivity (correctly predicted dropout). The average accuracy and sensitivity on the test set were 77% and 69%. Results are validated by a larger data set from several degree programs at another university. This study only used academic data to develop the model. Grades, course participation, and the rate of failed or unattended courses during the first semester were the most significant predictor of dropout risk. Huynh-Cam et al. [10] sought to equip universities with valuable insights to implement early

interventions aimed at reducing dropout rates. Their study utilized enrollment records from 2,412 first-year students at a private university in Taiwan. The researchers tested various machine learning models, including decision trees, logistic regression, and neural networks. Among these, the decision tree model outperformed other models, achieving an accuracy of 97.59%. Key predictive factors identified included student loans, father's occupations, mother's educational level, department, type of admission, school fee waivers, and primary sources of living. Kocsis et al. [2] reviewed and evaluated 95 studies published after 2012. They did a thorough comparison of data mining algorithms that have been applied to educational data sets, and they found that decision tree, logistic regression and neural networks are most commonly used educational data mining algorithms. Grade point average, obtained credits and gender were found to be most decisive predictors of academic performance. Silva, et al. [7] carried out a systematic review of 52 articles and examined various demographic, academic and learning factors that influence students' retention and dropout in the higher education system. In their study, they pointed out the multi-causal nature of university dropout. The most predictive factors were students' average scores, gender, course grades, course, age, ethnicity and scholarship. With these identified factors, they planned a machine learning project for the University of Brasilia to help improve retention rates."

- *Experiments, statistics, and other analyses are performed to a high technical standard and are described in sufficient detail.*

  Regarding this point, I believe the authors have done excellent work. The article presents a clear methodology, consistent with reference articles in the field. Statistical analyses are conducted at a high technical level and are described with sufficient detail.

  However, I note that the database used is somewhat outdated, covering the period from 2008/2009 to 2018/2019. Given that more than five years have passed since the collection of these data, the conclusions may no longer fully reflect current conditions.

  Response: This is the most recent open-source university dropout data set we can find. Although five years have passed since its collection, the framework of this study is applicable to more recent dropout data sets when they are available. Conclusions may be different. But at least for data sets with a similar data structure or a set of predictors, future researchers can use the findings of our study as a reference. We include this point as one limitation of our study in the new Discussion section as "Moreover, the data used in this research is five years old. While the conclusions provide valuable insights, they may not fully capture current trends. Nonetheless, the analytical framework presented in this study can be adapted to more recent datasets with similar structures or predictor sets. Future researchers may build upon this framework to examine updated data and explore the causal impacts of additional factors, potentially using methods like propensity score matching to enhance causal inference."

- *The conclusions are appropriately presented and supported by the data. The article successfully addresses its research objectives. However, the conclusions could be expanded, as not all results obtained are discussed.*

  The results section begins with a comparison of the evaluation of each statistical method used (Table 3), an important value-added aspect of the study. This helps readers better understand the statistical methodology employed.

  In this case, the potential of Table 4 is not fully explored, as the discussion is based on only two or three variables, leaving others unaddressed, neither in the conclusions nor in the broader analysis (for example, the article barely touches on academic variables and their effect on school dropout, even though these variables are identified as the second most explanatory factor in the model).

The conclusions section should be expanded to include all results obtained from the study. I believe the authors have much more information to offer readers.

Response: We expanded the Conclusion section, especially according to the contents included in Table 4. The new Conclusion section is written as below: "In this study, we analyze a data set compiled from a public university in Portugal, trying to predict college students' dropout using socioeconomic and academic features. Before modeling, we pre-process the features carefully, including multicollinearity checking, feature screening and propensity score matching, which is a crucial step to derive causal conclusions regarding the predictor `scholarship holder`. Several models demonstrate strong predictive validity. Random forest has the highest AUC value 0.937, effectively separating dropout students from graduated students. Random forest, XGBoost and GAM have close F1-scores, which are all around 0.9, showing good performance detecting dropout cases. Among the top seven important features found by XGBoost, three are relevant to students' financial situation: `scholarship holder`, `debtor` and `tuition fees up to date`. Using propensity score matching to balance covariates, our analysis with the GAM model reveals that being a scholarship holder significantly reduces the odds of dropping out, by nearly 40%. Table 4 highlights two additional financial factors strongly associated with dropout risk. First, owing money to a debtor is linked to a considerably higher risk, representing the largest effect among all covariates—a sixfold increase in the odds of dropping out. Second, timely payment of tuition fees is associated with a lower risk of dropping out. Displacement from residence also has a negative impact, although it remains unclear whether this is directly tied to students' financial situations. Beyond financial factors, academic and demographic variables play important roles. For instance, students enrolled in business programs face a higher dropout risk compared to those in health or human services programs. Application mode is another significant factor, with students applying during the second or third phases showing an increased likelihood of dropping out. Gender also influences dropout risk, with male students being more likely to leave their studies. Furthermore, marital status and parents' educational backgrounds are influential: single and never-married individuals face a higher risk, while students whose mothers are currently students themselves are more likely to drop out. However, we must exercise caution when interpreting factors other than being a scholarship holder. While these factors are significantly associated with the outcome, causal conclusions cannot be made because confounding effects are not eliminated."

- *The article presents some challenges in the presentation of tables and figures. There is a numbering overlap, with the same numbers assigned to tables within the text and to those in the appendix. Additionally, the figures in the appendix are not numbered. When the authors refer to these figures, they use terms such as "left" and "right," whereas assigning figure numbers would make referencing much clearer. If there are figures that are not referenced in the text, they should not be included in the manuscript or its appendix.*

  Response: We fixed the numbering overlap problem. The figures and tables in the appendix are now correctly numbered according to the journal's guideline. We changed Figure 3 to be two separate figures. S1_Figure in the appendix is also separated into three figures. Each of them has a unique label to refer to. Besides, we add a plot for the confusion matrix of the XGboost model, which is recommended by the Editor.

- *The research adheres to all applicable standards for ethical experimentation and research integrity.*

  Response: Yes. We agree.

Referee 2's Comments

- *The manuscript describes the use of multiple predictive models (Lasso, Random Forest, XGBoost, GAM, and Neural Networks) to analyze dropout risk. However, there is no clear rationale for why these specific models were chosen over others, nor is there a thorough discussion of the existing literature on using these models for similar purposes. It is essential for the authors to situate their approach within the context of existing studies. For example, are there other studies that have used these models to predict dropout risk among university students? What are the strengths and limitations of these models as reported in the literature? Furthermore, there are known controversies and debates within the literature regarding the performance of these models in various contexts, which have not been adequately addressed. A more comprehensive literature review would help clarify why the selected models are appropriate for this analysis.*

  Response: At the beginning of Section 4, we wrote the following to point out the advantage of each model "Lasso and GAM models are parametric and semi-parametric, which provides coefficient estimates for features and we use them to gain insights into the nature of the relationship between important features and the outcome. Random forest, XGBoost and artificial neural network are machine learning models, which usually have good prediction accuracy but lacks interpretability."

  To explain the advantage and disadvantage of each method and explain why we choose the models thoroughly, we add the following in the Introduction section at Line 109 to Line 131: "The predictive strength of each model largely depends on the dataset, which often cannot be determined in advance. In this study, we assess and compare the performance of five models: random forest, XGBoost, neural networks, Lasso, and generalized additive models (GAM). The first three models have been explored in previous research, consistently demonstrating high prediction accuracy. Among machine learning methods, tree-based ensemble approaches like random forest and boosting models are particularly notable for their predictive power and resistance to overfitting, especially with large datasets. These methods also offer the advantage of variable importance rankings, enabling researchers to identify the most influential predictors. Neural networks are powerful for capturing intricate patterns but require careful tuning to avoid overfitting. While these methods are widely appreciated for their accuracy, they are often seen as "black boxes," making interpretation difficult [3, 4]. Their lack of interpretability can hinder actionable insights, especially in fields like education where understanding the factors behind predictions is critical for intervention design. Lasso (Least Absolute Shrinkage and Selection Operator) [5] offers improved interpretability and a lower risk of overfitting compared to logistic regression, which is a method frequently employed in prior studies. However, Lasso is less capable of capturing intricate nonlinear relationships compared to tree-based models or neural networks. Generalized additive model (GAM) [6] strikes a balance between interpretability and flexibility. While GAMs may not have as high prediction accuracy as tree-based methods or neural networks on, their semiparametric nature makes them valuable for providing insights into how specific predictors influence dropout risk. This feature is particularly beneficial for actionable research in fields like education."

- *Please make the aim of the study clear in the introduction.*

  Response: After we finish the literature review, we add the following in order to highlight the cons of previous studies and introduce our objectives: "The goal of this study is as follows: by analyzing a data set with 4,424 students enrolled in 17 undergraduate programs at a public university in Portugal, we seek to strengthen and complement the findings of previous studies. More importantly, we aim to explore the nature of the relationships between key features and dropout status, with a particular focus on understanding the causal link between scholarship status and the risk of dropping out. While previous studies have identified various factors that contribute to dropout risks, they have seldom explored causal inferences. Furthermore, many of these factors—such as age, ethnicity,

and parental background—are beyond the control of universities. In contrast, scholarships represent an intervention that universities can manage, making it essential to examine how receiving a scholarship influences the likelihood of dropping out. We apply propensity score matching to draw a conclusion about the causal link between scholarship status and dropout risk. Additionally, even for covariates—particularly categorical ones—where causal inference is not the focus, it is valuable to quantify and interpret their effect on droppping out. However, this has not been fully discussed in previous studies. For example, although Ortiz-Lozano et al. [8] used decision trees for their interpretability, the model did not provide insight into how the outcome changes as predictors vary. Similarly, while some studies reviewed by Kocsis et al. [2] or Huynh-Cam et al. [10] employed logistic regression for its linear interpretation, their models lacked variable selection, which led to issues of overfitting and multicollinearity, undermining their interpretability. Moreover, when the relationships are highly non-linear, the linear interpretation is not valid anymore. To achieve better interpretation of covariates, we apply a semiparametric model, an approach not discussed in prior research."

- *Lines 2-6: Please include references to support this explanation: Understanding important factors in predicting college dropouts is not only important to colleges and universities, but also to a nation. To colleges and universities, finding ways to mitigate college dropout rates can lead to better utilization of funds for student resources. To a nation, mitigating college dropout rates can potentially lead to a lower unemployment rate and an increase in the nation's workforce.*

  Response: We removed these lines and re-write the beginning of the Introduction section as

  Understanding important factors that are associated with student dropouts from higher education is important to institutions and students. Colleges and universities have the responsibility to help students succeed. High dropout rates indicate that many students are not receiving the support they need, whether academically or financially. Detecting the factors can allow institutions to act early to control or reduce dropout rates, help students get a degree, and pave the way for a better career outlook. According to the Education Data Initiative [1], college dropouts are 20% more likely to be unemployed compared to those who complete their degrees.

  The following references are included to support the statement:

  1. Silva, E. C., Freitas, S., Soares Ramos, C., Muniz De Menezes, A. E., & Rodrigues De Araujo, L. K. S. (2023). A systematic review of the factors that impact the prediction of retention and dropout in higher education.

  2. Ortiz-Lozano, J. M., Aparicio-Chueca, P., Triadó-Ivern, X. M., & Arroyo-Barrigüete, J. L. (2024). Early dropout predictors in social sciences and management degree students. Studies in Higher Education, 49(8), 1303-1316.

  3. Ortiz-Lozano, J. M., Rua-Vieites, A., Bilbao-Calabuig, P., & Casadesús-Fa, M. (2020). University student retention: Best time and data to identify undergraduate students at risk of dropout. Innovations in education and teaching international.

  4. Huynh-Cam, T. T., Chen, L. S., & Lu, T. C. (2024). Early prediction models and crucial factor extraction for first-year undergraduate student dropouts. Journal of Applied Research in Higher Education.

- *The manuscript fails to provide sufficient information about the study variables, which makes it difficult to understand, follow, and replicate the study. The authors mention that 36 variables were used in the analysis but do not explain what these variables are, how they were collected, or whether they include covariates.*

  Response: We did provide such information. At Line 67 to Line 69 in the Introduction section, we said that the data was collected and first discussed by researchers at a university in Portugal [11],

which includes steps about how the data set was collected. We provided the data source as well. In the Data preprocessing section, i.e., Section 3.2, we explained how we preprocess the main predictor and covariates, which predictors are not used, why they are not used, etc. Finally, at Line 196, we pointed out to the reader that continuous and categorical variables for this study are summarized in two tables in the Appendix.

- *One of the aims of the study is to understand the causal effect of being a scholarship holder on dropout rates. However, the results presented in the manuscript do not provide clear insights into this relationship. There is no discussion on how the authors have addressed potential confounding factors, nor is there any robust analysis to determine causality.*

  Response: We did. The analysis is called propensity score matching, which is a tool used to balance distributions of covariates in a "control group" and a "treatment group", and derive a valid causal conclusion for the main predictor of interest. Details were included in Section 3.2.5.

- *The manuscript lacks a dedicated discussion section, which significantly weakens its contribution to the field. A discussion section is essential for interpreting the results, situating the findings within the broader context of the literature, and highlighting the practical implications of the study. Furthermore, it provides an opportunity for the authors to acknowledge the limitations of their work and suggest directions for future research. In the current manuscript, the results are presented without sufficient interpretation.*

  Response: We add a Discussion section. The new Discussion section is as below:

  "To our best knowledge, this study has several limitations. First, the Polytechnic Institute of Portalegre is a public university, and the factors influencing student dropout rates may vary between public and private institutions. Second, every individual used in this study has information on the qualifications and occupations of both parents. However, the impact of parental background could be different in single-parent families, and this is worth discussing in future research. Additionally, some variables in the dataset lack clear definitions. For example, while the `application mode` was identified as a significant predictor, but authors of [11], who collected the data set did not provide details on the three phases of this variable. Moreover, the data used in this research is five years old. While the conclusions provide valuable insights, they may not fully capture current trends. Nonetheless, the analytical framework presented in this study can be adapted to more recent datasets with similar structures or predictor sets. Future researchers may build upon this framework to examine updated data and explore the causal impacts of additional factors, potentially using methods like propensity score matching to enhance causal inference. Finally, dropout factors are likely to vary across countries. Future researchers could investigate the impact of national context on university dropout rates. In summary, this study reinforces findings from prior research, confirming that university dropout is shaped by a variety of factors, including financial, academic, and demographic influences. By employing propensity score matching, this study identifies the causal impact of holding a scholarship on dropout rates. Additionally, it quantifies the effects of several covariates that have not been thoroughly examined in earlier studies, contributing new insights to the literature on dropout risk factors."

  The Conclusion section is expanded such that more in-depth interpretation of the results is included. The new Conclusion section is as below:

  "In this study, we analyze a data set compiled from a public university in Portugal, trying to predict college students' dropout using socioeconomic and academic features. Before modeling, we pre-process the features carefully, including multicollinearity checking, feature screening and propensity score matching, which is a crucial step to derive causal conclusions regarding the predictor

`scholarship holder`. Several models demonstrate strong predictive validity. Random forest has the highest AUC value 0.937, effectively separating dropout students from graduated students. Random forest, XGBoost and GAM have close F1-scores, which are all around 0.9, showing good performance detecting dropout cases. Among the top seven important features found by XGBoost, three are relevant to students' financial situation: `scholarship holder`, `debtor` and `tuition fees up to date`. Using propensity score matching to balance covariates, our analysis with the GAM model reveals that being a scholarship holder significantly reduces the odds of dropping out, by nearly 40%. Table 4 highlights two additional financial factors strongly associated with dropout risk. First, owing money to a debtor is linked to a considerably higher risk, representing the largest effect among all covariates—a sixfold increase in the odds of dropping out. Second, timely payment of tuition fees is associated with a lower risk of dropping out. Displacement from residence also has a negative impact, although it remains unclear whether this is directly tied to students' financial situations. Beyond financial factors, academic and demographic variables play important roles. For instance, students enrolled in business programs face a higher dropout risk compared to those in health or human services programs. Application mode is another significant factor, with students applying during the second or third phases showing an increased likelihood of dropping out. Gender also influences dropout risk, with male students being more likely to leave their studies. Furthermore, marital status and parents' educational backgrounds are influential: single and never-married individuals face a higher risk, while students whose mothers are currently students themselves are more likely to drop out. However, we must exercise caution when interpreting factors other than being a scholarship holder. While these factors are significantly associated with the outcome, causal conclusions cannot be made because confounding effects are not eliminated."

- *The limitations of the study, which should be included in the discussion, are part of the conclusion.*

  Response: We add a Discussion section and move the limitation to be in the Discussion section.

Editor's Comments

- *There is a significant lack in the literature review. Please update a section for a literature.*

  Response: We expanded the introduction section of the paper with a more comprehensive literature review. The new references we include are:

  1. Silva, E. C., Freitas, S., Soares Ramos, C., Muniz De Menezes, A. E., & Rodrigues De Araujo, L. K. S. (2023). A systematic review of the factors that impact the prediction of retention and dropout in higher education.

  2. Ortiz-Lozano, J. M., Aparicio-Chueca, P., Triadó-Ivern, X. M., & Arroyo-Barrigüete, J. L. (2024). Early dropout predictors in social sciences and management degree students. Studies in Higher Education, 49(8), 1303-1316.

  3. Ortiz-Lozano, J. M., Rua-Vieites, A., Bilbao-Calabuig, P., & Casadesús-Fa, M. (2020). University student retention: Best time and data to identify undergraduate students at risk of dropout. Innovations in education and teaching international.

  4. Huynh-Cam, T. T., Chen, L. S., & Lu, T. C. (2024). Early prediction models and crucial factor extraction for first-year undergraduate student dropouts. Journal of Applied Research in Higher Education.

  5. Robert Tibshirani. (1996). Regression Shrinkage and Selection via the Lasso. Journal of the Royal Statistical Society, Series B.

  Besides, for references we included in the first version of the paper, we discuss them with more details.

- *At the end of the Lit, make sure to highlight the cons of the past studies and introduce your objectives.*

  Response: After we finish the literature review, we add the following in order to highlight the cons of previous studies and introduce our objectives: "The goal of this study is as follows: by analyzing a data set with 4,424 students enrolled in 17 undergraduate programs at a public university in Portugal, we seek to strengthen and complement the findings of previous studies. More importantly, we aim to explore the nature of the relationships between key features and dropout status, with a particular focus on understanding the causal link between scholarship status and the risk of dropping out. While previous studies have identified various factors that contribute to dropout risks, they have seldom explored causal inferences. Furthermore, many of these factors—such as age, ethnicity, and parental background—are beyond the control of universities. In contrast, scholarships represent an intervention that universities can manage, making it essential to examine how receiving a scholarship influences the likelihood of dropping out. We apply propensity score matching to draw a conclusion about the causal link between scholarship status and dropout risk. Additionally, even for covariates—particularly categorical ones—where causal inference is not the focus, it is valuable to quantify and interpret their effect on droppping out. However, this has not been fully discussed in previous studies. For example, although Ortiz-Lozano et al. [8] used decision trees for their interpretability, the model did not provide insight into how the outcome changes as predictors vary. Similarly, while some studies reviewed by Kocsis et al. [2] or Huynh-Cam et al. [10] employed logistic regression for its linear interpretation, their models lacked variable selection, which led to issues of overfitting and multicollinearity, undermining their interpretability. Moreover, when the relationships are highly non-linear, the linear interpretation is not valid anymore. To achieve better interpretation of covariates, we apply a semiparametric model, an approach not discussed in prior research."

- *You can add more illustrations to show the results, ex: Confusion matrix for predictions maybe?*

  Response: We add a confusion matrix for the XGBoost model, which can be considered as the optimal model according to evaluation metrics in Section 4.7. A sentence is added at Line 341 to Line 344 as "The confusion matrix in Fig 3 illustrates the predictive performance of the XGBoost model on the test set. Rows of this matrix represent the cases classified into the dropout and graduate groups, while the columns indicate the actual group membership of each case."

References

[1] Hanson Melanie. College dropout rates. 2023. Available from: `https://educationdata.org/college-dropout-rates`.

[2] Ádám Kocsis, Gyöngyvér Molnár. Factors influencing academic performance and dropout rates in higher education. Oxford Review of Education.2024. doi:10.1080/03054985.2024.2316616.

[3] Breiman L. Random forests. Machine Learning. 2001;45(1);5–32. doi:10.1023/A:1010933404324.

[4] Chen T, Guestrin C. XGboost: a scalable tree boosting system. In: Proceedings of the 22nd ACM SIGKDD International Conference on Knowledge Discovery and Data Mining; 2016. p. 785–794. doi:10.1145/2939672.2939785.

[5] Robert Tibshirani. Regression Shrinkage and Selection via the Lasso.1996;58(1);267–288. Journal of the Royal Statistical Society, Series B. doi:10.1111/j.2517-6161.1996.tb02080.x.

[6] Hastie T, Tibshirani R, Friedman J. The Elements of Statistical Learning: Data mining, Inference and Prediction. 2nd ed. New York, NY, USA: Springer; 2009.

[7] Cosme da Silva Edmilson, Freitas Sergio, Ramos Cristiane, Muniz de Menezes Amanda, Araújo Letícia. A systematic review of the factors that impact the prediction of retention and dropout in higher education. doi:10.24251/HICSS.2023.157.

[8] José María Ortiz-Lozano, Antonio Rua-Vieites, Paloma Bilbao-Calabuig, Martí Casadesús-Fa. University student retention: best time and data to identify undergraduate students at risk of dropout. Innovations in Education and Teaching International. 2020; 57(1):74–85. doi:10.1080/14703297.2018.1502090.

[9] José M. Ortiz-Lozano, Pilar Aparicio-Chueca, Xavier M. Triadó-Ivern, Jose Luis Arroyo-Barrigüete. Early dropout predictors in social sciences and management degree students. Studies in Higher Education.2024;49(8):1303–1316. doi:10.1080/03075079.2023.2264343.

[10] Thao-Trang Huynh-Cam, Long-Sheng Chen, Tzu-Chuen Lu. Early prediction models and crucial factor extraction for first-year undergraduate student dropouts.2024. Vol. ahead-of-print No. ahead-of-print. Journal of Applied Research in Higher Education. doi:10.1108.JARHE-10-2023-0461.

[11] Martins Mónica V., Tolledo Daniel, Machado Jorge, Baptista Luís M. T., Realinho, Valentim. Early prediction of student's performance in higher education: a case study. Advances in Intelligent Systems and Computing Trends and Applications in Information Systems and Technologies. 2021;166–175. doi:10.1007/978-3-030-72657-7_16.
