# Supplementary material for: Statistical and machine learning models for predicting university dropout and scholarship impact
Source: PLoS One. 2025 Jun 25;20(6):e0325047. doi: 10.1371/journal.pone.0325047 (PMC12193850; doi:10.1371/journal.pone.0325047)
Supplement: Attachment — Submitted filename: responses_to_reviewers_R2.pdf [file pone.0325047.s007.pdf]

Responses to Referee's and Editor's Comments

We the authors are very thankful to the Referee and the Editor for their suggestions and comments, which we believe greatly help improve the presentation of this paper.

Editor's Comments

1. *The abstract provides a concise summary of the study's objectives, methodology, and key findings. However, it could be improved by clearly defining the term "dropout" and specifying the types of degrees considered (undergraduate, master's, or both). Additionally, the abstract should briefly mention the limitations of the study to provide a balanced overview.*

   Response: We add the definition of dropout to the abstract as "In this study, dropout is defined from a micro-perspective, where field and institution changes are considered as dropouts independently of the timing these occur." We add the following sentence to briefly mention the limitation "As the study is based on data from a single institution and time period, and unobserved confounders cannot be fully ruled out, results should be interpreted with caution."

2. *The introduction effectively highlights the importance of understanding dropout factors. However, it lacks a comprehensive literature review. The authors should include more recent studies and discuss the strengths and limitations of various predictive models used in similar research. This will help situate their work within the broader context of existing literature.*

   Response: We cite the following recent studies, which are recommended by Referee 3, in the Introduction Section. The new paragraph is:

   "Aina et al. [1] did a comprehensive review of socio-economic literature for the past three decades on the student determinants of tertiary education dropout and provided insights about the main trends in university dropouts. By synthesizing empirical findings within a theoretical framework, the authors examined how informative issues (emphasized by the economic literature) and relational elements (emphasized by the sociological literature) contribute to students' decisions to discontinue their tertiary education. The factors include student demographic characteristics, abilities and behavior, parental background and family networks, academic/social integration and institutional/goal commitment, tuition fees and financial/in-kind aid, etc. Logistic regression is the most commonly used model in the studies reviewed in this paper. Empirical evidence showed that older freshmen, minority students, and students from poorer families have a higher dropout probability. On the other hand, female students, students with closer ties with peers, and students in institutions with greater commitment are less likely to drop out. Higher tuition fees only affect low-income students and financial aid has a positive impact on student retention. Lorenzo-Quiles et al. [2] is another recent review study, which used different databases to find out the most appropriate authors on the topic of university dropout since 2018 and identify variables that can cause university dropouts. Identified causes are mainly psychological and social factors. Support from parents, teachers and institutions is often one determinant why students regain their motivation and stay in institutions rather than drop out. Self-esteem and self-concept that lead to school failure or frustration are also directly related to dropout. Venkatesan et al. [3] reviewed 36 studies publish between 2000 to 2023, which applied statistical and machine learning approaches to predict student dropout in education. It was found that Random Forest and Decision Tree are among the most widely used techniques in those recent studies. Prediction accuracy and Area Under the Curve (AUC) are the most frequently employed metrics to assess the performance of predictive models. This review also pointed out some unsolved

problems such as data imbalance, lack of interpretability of machine learning models, and geographic disparities that might lead to new research in the future."

We add the following paragraph to discuss the strength and limitation of current popular models, and present the research gap we aim to address in this study:

"The research gap we aim to address centers on three aspects: 1) estimating causal effects of scholarship status on dropout using propensity score matching (PSM), 2) improving model interpretability in dropout prediction, and 3) evaluating the performance and utility of boosting methods, which remain under-explored in recent literature on student dropout. While prior studies have identified factors associated with dropout risk, few have investigated the causal relationship between financial support and dropout. By applying PSM, we seek to isolate the effect of holding a scholarship, thereby moving beyond correlation-based findings. In addition, we explore the potential of machine learning models—particularly the Least Absolute Shrinkage and Selection Operator (Lasso) [4], Generalized Additive Models (GAMs) [5] and XGBoost [6] — to offer both interpretability and predictive strength. Logistic regression was commonly used in previous studies due to its ease of interpretation. However, when applied to datasets with a large number of predictors—as is often the case in dropout studies—logistic regression becomes susceptible to multicollinearity and overfitting, which can compromise both model performance and interpretability. The Lasso method is a shrinkage method to reduce the multicollinearity and overfitting problem in logistic regression. We explore the Lasso method to evaluate whether it can achieve both high predictive accuracy and maintain a transparent linear structure. GAM is a more flexible model to handle non-linear effects than Lasso. As a semi-parametric method, GAM strikes a balance of flexibility and interpretability. We further demonstrate that boosting methods like XGBoost can yield high predictive performance and valuable insights into variable importance like Random Forest, offering a complementary perspective to traditional statistical approaches. Our study, based on data from 4,424 students enrolled in 17 undergraduate programs at a public university in Portugal, thus contributes substantively to the understanding of dropout dynamics."

3. *Definition of dropout. Clearly define what constitutes a dropout. Are students who switch majors or institutions considered dropouts? This distinction is crucial for the study's validity.*

   Response: We add the following paragraph in Section 3.1:

   "In this study, dropout is defined from a micro-perspective, where field and institution changes are considered as dropouts independently of the timing these occur. In contrast, a macro-perspective might define dropout more leniently. For example, only students who leave the entire education system and won't return are considered as dropouts, and students who switch majors or transfer schools are still considered enrolled, etc."

4. *Propensity Score Matching (PSM). Justify the choice of the "subclass" method over other methods like "nearest neighbor." Address recent criticisms of PSM and consider alternative methods like Coarsened Exact Matching (CEM).*

   Response: The "subclass" method in the MatchIt package represents the stratification method on the propensity score. It estimates propensity scores, creates strata and balances covariates within each stratum. We chose "subclass" over "nearest neighbor" because of better matching results, as indicated by diagnostic measures shown in Fig 3.. We evaluated covariate balance before and after matching using standardized mean differences (SMDs) and variance ratios. After matching, nearly all covariates had absolute SMDs below 0.1, and most variance ratios fell within the acceptable range of 0.8 to 1.25. These results suggest that the subclass method effectively reduced selection bias in our context. As for CEM, while it offers the advantage of exact matching within coarsened strata,

determining the appropriate level of coarsening for each covariate is itself a complex and subjective task, Besides, we want continuity for variables such as age for interpretability in the modeling stage. We consider Regression Discontinuity Design (RDD) as an alternative quasi-experimental method.

We add two paragraphs in Section 3.2.5. The new paragraphs are as:

"In this study, a randomized controlled trial (RCT) is not feasible or inappropriate. For example, if a scholarship is aimed to be provided to students from low-income families or minority groups, then randomly issuing or denying the scholarship to qualified students would raise ethical concerns. Therefore, we find it necessary to employ a quasi-experimental approach to avoid ethical issues of random assignment, such as propensity score matching, to mimic the effects of RCT and to explore the influence of scholarship on dropout risks."

"To choose among the options, we assess covariate balance before and after matching using standardized mean differences (SMDs) and variance ratios. The matching method that results in the best balance is the "subclass" method. As shown in Fig 1., matching substantially reduced imbalance: after matching, nearly all covariates had absolute SMDs below 0.1, and variance ratios were within an acceptable range (most between 0.8 and 1.25). These diagnostics indicate that our matching procedure achieved strong balance between treated and control groups, improving the credibility of the causal estimates. Details of theoretical support of these diagnostic tools can be found in [9]. However, we want to caution that when using propensity score matching, unobserved confounders, such as family expectations or mental health, may bias causal estimates if they influence both scholarship receipt and dropout risk. Regression Discontinuity Design (RDD) proposed in [7] offers a viable alternative, as it relies on a known cutoff for treatment assignment and assumes local similarity around the threshold. For example, if a scholarship is awarded based on income, RDD can estimate the causal effect by comparing students just below and above the income cutoff. This method avoids reliance on full covariate adjustment and may better approximate a randomized experiment. A relevant case study discussing the causal effect of financial aid on university attrition can be found in [8]. Future research could explore such designs in more detail using richer datasets. "

5. *Confounding variables. Specify the confounding variables included in the PSM. Ensure these variables affect both the outcome (dropout) and the treatment (scholarship status).*

   Response: We add the following paragraph in Section 3.2.5:

   "The following variables covariates were included in the propensity score estimation 1) demographic factors: age at enrollment, gender, marital status, displaced status (whether the student relocated from their residence to attend the university) 2) academic background: previous qualification, previous qualification grade, admission grade, application order 3) academic performance and engagement: curricular units enrolled, credited, evaluated, and graded across semesters; curricular units without evaluations 4) socioeconomic indicators: tuition fee payment status, debtor status, mother's and father's occupations 5) administrative and institutional factors: course of study, daytime/evening attendance, application mode. These covariates were selected based on their practical relevance and prior empirical evidence showing associations with both scholarship allocation and dropout risk [1,3]. Academic performance indicators (e.g., admission grade, prior qualifications, course grades) are typically used in scholarship decisions and are strong predictors of academic persistence. Socioeconomic variables such as tuition payment status, debtor status, and parental occupations reflect financial stability, which may influence both eligibility for financial aid and the ability to continue education. Demographic and institutional factors—including age, gender, course type, and attendance mode—are also known to correlate with both the likelihood of receiving support and the risk of dropout. Together, these factors represent key dimensions (academic, economic, and demographic) commonly linked to both treatment and outcome in higher education research."

6. *Quantify the importance of features identified by XGBoost. Explain how each feature influences the dropout risk.*

   Response: We add Table 4. in Section 5.2 showing importance scores of each important predictor. We add the following sentence to explain how the importance scores are computed: "The boosting model computes feature importance by summing the reduction in the model's binary entropy loss function caused by splits on each feature across all trees. Features with higher importance scores have a greater impact on the model's performance."

7. *Provide a more detailed interpretation of GAM results. Use marginal effects to explain the impact of significant predictors like scholarship status and debt on dropout probability.*

   Response: We add a column in Table 5. in Section 5.3.1 showing the marginal effects (ME) of significant predictors, which show the change in predicted probability of dropping out between levels of a categorical variable, when holding other predictors as constant. Interpretation is included in the new Conclusion Section, which is shown in bullet 10. below.

8. *Include a confusion matrix for the XGBoost model to illustrate its predictive performance.*

   Response: We visualize the confusion matrix for the XGBoost model in Fig 4. in Section 5.2. We add the following sentences to explain it. "The confusion matrix in Fig 4. illustrates the predictive performance of the XGBoost model on the test set. Rows of this matrix represent the cases classified into the dropout and graduate groups, while the columns indicate the actual group membership of each case."

9. *The discussion section is currently missing. This section should interpret the results, compare them with findings from other studies, and discuss the practical implications for universities. Highlight the limitations of the study and suggest directions for future research.*

   Response: We write a new Discussion Section, and switch the order of this section and the Conclusion Section, according to the editor's advice. **The new Discussion Section, which highlights limitations and future research is as below:**

   "This study contributes to the literature on student dropout by integrating machine learning models with quasi-experimental analysis to evaluate both associative patterns and potential causal effects, particularly concerning financial aid. The results consistently identified financial hardship, academic disengagement, and student background characteristics as key predictors of dropout. The importance of financial vulnerability was strongly supported by both the GAM model and XGBoost feature importance. Being in debt, having unpaid tuition fees, and lacking a scholarship emerged as the most predictive financial variables—aligning closely with recent findings that emphasize financial instability as a major driver of student dropout [1, 3, 8]. Our analysis using propensity score matching also showed that receiving a scholarship significantly reduces the likelihood of dropout, echoing prior research that highlights the retention benefits of financial support [2,8]. These results reinforce ongoing calls for institutions to prioritize financial aid accessibility, scholarship expansion, and debt management programs as central to student success strategies. Academic engagement was another critical dimension of dropout risk. Students failing to complete second-semester courses were substantially more likely to leave their studies – aligning with findings from [10], which emphasized that predictive models based on first-year performance data can help identify high-risk students early in their academic journey. From a policy perspective, the implications for universities are clear: tracking academic and financial indicators in real time can allow institutions to identify students at risk and intervene before dropout occurs. For example, early alerts based on tuition payment status, course completion rates, or accumulated debt could initiate timely outreach through advising, tutoring, or

emergency financial aid. This aligns with [2], who advocate for data-informed strategies that address both academic and socio-economic vulnerabilities.

This study has several limitations. First, the dataset used is five years old, and while it provides a robust historical overview, recent changes in higher education, such as remote learning or post-pandemic effects, may affect the generalizability of our findings. Second, the data were collected from a single public institution in Portugal. Institutional characteristics such as funding models, admissions policies, and student demographics differ widely across universities and countries. Thus, caution is warranted when generalizing these results to other contexts, especially outside of Portugal or within private institutions. Third, while propensity score matching adjusts for observed confounders, it cannot eliminate bias from unmeasured factors such as motivation, mental health, family pressure, or institutional support systems. These unobserved variables may influence both scholarship receipt and dropout, limiting the strength of causal inference. Finally, although the GAM model allowed for interpretability, it does not capture all possible interactions among important predictors.

In the future, improvements could be made by integrating data from multiple institutions or applying alternative causal inference techniques such as regression discontinuity design (RDD). RDD may be especially useful in settings where scholarship eligibility is based on a clear income or academic threshold, offering stronger quasi-experimental validity, as shown in [8]. Additionally, some potentially relevant variables, such as mental health, social integration, or employment status [2], were not available in our dataset. Integrating such information on student experiences could complement and enrich model-driven insights. When working with extremely large datasets, deep learning models, such as neural networks, may outperform GAM or XGBoost in predictive accuracy due to their ability to capture complex, nonlinear interactions among variables. Future researchers might also apply emerging techniques from explainable AI (XAI), such as SHapley Additive exPlanations [11], to help improve interpretability of deep learning models. This could allow institutions to benefit from both the accuracy of neural networks and the transparency needed for actionable insights."

10. *The conclusion summarizes the key findings but should be expanded to include a discussion of all significant results. Address the implications of financial and academic factors on dropout rates and suggest specific interventions that universities can implement.*

    Response: We write a new Conclusion Section as follows:

    "This study evaluated five statistical learning models for predicting university dropout, using a dataset collected from a public university in Portugal. Among the models tested, XGBoost demonstrated the highest overall performance (Accuracy = 0.885, F1 = 0.909, Recall = 0.875, Precision = 0.947). Key predictors of dropping out identified by XGBoost included the number of completed curricular units, up-to-date tuition payments, age at enrollment, and scholarship status. While Random Forest achieved the highest AUC (0.937), the AUC scores of the other models were comparably strong. The Generalized Additive Model (GAM) performs competitively in terms of standard evaluation metrics. More importantly, it offered clear interpretability, revealing how changes in actionable variables influence dropout risk. The findings confirm that both financial and academic factors are central to predicting student attrition. For example, students with unpaid tuition or existing debt face significantly higher dropout risk: being a debtor increases the odds of dropping out nearly sixfold (OR = 5.92), or equivalently, increases the probability of dropout by 35.3%, holding other factors constant. Conversely, students who keep their tuition fees up to date experience a 42.4% reduction in dropout probability. Using propensity score matching to adjust for covariate imbalance, the model also shows that receiving a scholarship leads to the reduction in the odds of dropping out by nearly 40%, or by 22.2% in terms of probability. Beyond financial variables, academic and demographic factors also play a substantial role. Students enrolled in business programs face an 18.8% higher risk

of dropping out compared to those in health or human services fields. Application timing is another important factor: students who apply during the second or third admission phases are more likely to leave their studies, exhibiting a 12.2% and 28.2% higher chance of dropping out. Being displaced was associated with a 12.8% increase in the likelihood of dropping out. Gender differences were observed as well, with male students exhibiting a 13.7% higher likelihood of dropping out. Marital status and parental education levels also influence dropout rates—single or never-married students are more at risk, and those whose mothers are currently students themselves show a stronger risk of student dropout. Among continuous variables, age at enrollment and the number of graded curricular units completed in the second semester emerged as the most influential predictors. Figs 5 and 6 illustrate their marginal effects. As shown in Fig. 5, the probability of dropout initially increases with age at enrollment, peaking around the median age of 20, and then declines. Fig. 6 highlights the heightened dropout risk among students demonstrating low academic engagement or performance.

Universities can act on these findings through targeted interventions. Early academic risk indicators, such as failing to complete second-semester courses, can trigger proactive advising or tutoring. Monitoring tuition status can help identify students in financial distress, who may benefit from emergency aid, payment plans, or financial counseling. Given the greater dropout risk among students without scholarships or those entering via later application phases, expanded scholarship access and tailored orientation programs could enhance student integration and persistence. Additionally, displaced students may require flexible academic policies and specialized support services. By combining accurate prediction with interpretable insights, this study offers a data-driven foundation for designing student support strategies that address both academic and financial vulnerabilities, which have been shown as two critical dimensions of university dropout. ”

11. *The limitations section should be more comprehensive. Discuss the potential impact of using outdated data and the generalizability of the findings to other institutions and countries. Acknowledge the limitations of the predictive models used and suggest improvements for future research.*

    Response: As recommended in bullet 9., we include limitations in the Discussion Section.

12. *Data visualization. Use more visual aids like graphs and charts to present the results. This will make the findings more accessible and engaging.*

    Response: According to the journal's policy, figures or any visualization cannot be included in the manuscript. They can only be submitted as separate files with the format to be .tiff. We provide the following separate figure files to illustrate the results and findings.

    - Fig 1. The file name is match_balance.tiff. It llustrates the balance of covariates after propensity score matching using the "subclass" method.

    - Fig 2. The file name is xgboost.tiff. It shows features' importance value generated by the XGBoost model.

    - Fig 3. The file name is xgb_confusionmatrix.tiff. It visualizes the confusion matrix for the XGBoost model being evaluated with the test set.

    - Fig 4. The file name is s5_plot_v2.tiff. It shows how the probability of dropping out changes when age at enrollment increases, while holding all other predictors as constant.

    - Fig 5. The file name is s10_plot_v2.tiff. It shows how the probability of dropping out changes when graded curricular units increases, while holding all other predictors as constant.

    - Fig 6. The file name is roc_curves.tiff. It shows the ROC curves for the 5 models used in this study.

13. *Practical applications. Provide concrete examples of how universities can use the study's findings to design interventions and support at-risk students.*

Response: As recommended in bullet 10., we address this point in the Conclusion Section.

Referee 1's Comments

All comments have been addressed.

Referee 2's Comments

All comments have been addressed.

Referee 3's Comments

1. *First, the topic addressed in the manuscript is highly relevant. Given the current challenges faced by universities due to demographic shifts and declining enrollment rates, it is crucial to analyze the academic trajectories of students to better support them throughout their educational journey. The study's focus on predicting university dropout is, therefore, well justified.*

   Response: Thanks. We agree.

2. *However, while the relevance of the topic is clear, certain aspects of the theoretical justification require further development. Specifically, the literature review lacks depth, and the manuscript does not clearly outline the main trends in university dropout. To strengthen the conceptual framework, the authors could consider incorporating key references such as Venkatesan et al. (2024), Aina et al. (2022), and Lorenzo-Quiles et al. (2023), which provide recent insights into this issue.*

   Response: We add a new paragraph to cite these references in the Introduction section as below:

   "Aina et al. [1] did a comprehensive review of socio-economic literature for the past three decades on the student determinants of tertiary education dropout and provided insights about the main trends in university dropouts. By synthesizing empirical findings within a theoretical framework, the authors examined how informative issues (emphasized by the economic literature) and relational elements (emphasized by the sociological literature) contribute to students' decisions to discontinue their tertiary education. The factors include student demographic characteristics, abilities and behavior, parental background and family networks, academic/social integration and institutional/goal commitment, tuition fees and financial/in-kind aid, etc. Logistic regression is the most commonly used model in the studies reviewed in this paper. Empirical evidence showed that older freshmen, minority students, and students from poorer families have a higher dropout probability. On the other hand, female students, students with closer ties with peers, and students in institutions with greater commitment are less likely to drop out. Higher tuition fees only affect low-income students and financial aid has a positive impact on student retention. Lorenzo-Quiles et al. [2] is another recent review study, which used different databases to find out the most appropriate authors on the topic of university dropout since 2018 and identify variables that can cause university dropouts. Identified causes are mainly psychological and social factors. Support from parents, teachers and institutions is often one determinant why students regain their motivation and stay in institutions rather than drop out. Self-esteem and self-concept that lead to school failure or frustration are also directly related to dropout. Venkatesan et al. [3] reviewed 36 studies publish between 2000 to 2023, which applied

statistical and machine learning approaches to predict student dropout in education. It was found that Random Forest and Decision Tree are among the most widely used techniques in those recent studies. Prediction accuracy and Area Under the Curve (AUC) are the most frequently employed metrics to assess the performance of predictive models. This review also pointed out some unsolved problems such as data imbalance, lack of interpretability of machine learning models, and geographic disparities that might lead to new research in the future."

Besides, we add another two citations, which are included in 4. to strengthen the methodological rigor when we apply propensity score matching.

3. *Additionally, the study's contribution needs to be more explicitly defined. It would be beneficial for the authors to clarify the specific research gap they aim to address and to better articulate what the use of machine learning offers compared to traditional techniques such as logistic regression. Expanding on this point would reinforce the manuscript's originality and its added value to the field.*

   Response: We write a new paragraph as follows:
   "The research gap we aim to address centers on three aspects: 1) estimating causal effects of scholarship status on dropout using propensity score matching (PSM), 2) improving model interpretability in dropout prediction, and 3) evaluating the performance and utility of boosting methods, which remain under-explored in recent literature on student dropout. While prior studies have identified factors associated with dropout risk, few have investigated the causal relationship between financial support and dropout. By applying PSM, we seek to isolate the effect of holding a scholarship, thereby moving beyond correlation-based findings. In addition, we explore the potential of machine learning models—particularly the Least Absolute Shrinkage and Selection Operator (Lasso) [4], Generalized Additive Models (GAMs) [5] and XGBoost [6] — to offer both interpretability and predictive strength. Logistic regression was commonly used in previous studies due to its ease of interpretation. However, when applied to datasets with a large number of predictors—as is often the case in dropout studies—logistic regression becomes susceptible to multicollinearity and overfitting, which can compromise both model performance and interpretability. The Lasso method is a shrinkage method to reduce the multicollinearity and overfitting problem in logistic regression. We explore the Lasso method to evaluate whether it can achieve both high predictive accuracy and maintain a transparent linear structure. GAM is a more flexible model to handle non-linear effects than Lasso. As a semi-parametric method, GAM strikes a balance of flexibility and interpretability. We further demonstrate that boosting methods like XGBoost can yield high predictive performance and valuable insights into variable importance like Random Forest, offering a complementary perspective to traditional statistical approaches. Our study, based on data from 4,424 students enrolled in 17 undergraduate programs at a public university in Portugal, thus contributes substantively to the understanding of dropout dynamics."

4. *Regarding the methodology, the authors should be cautious when discussing causality, as the study employs a quasi-experimental approach. The temporal sequencing and directionality of certain variables—such as scholarship allocation—are not clearly established. Since this is not a randomized controlled trial (RCT), the manuscript should acknowledge the limitations in making causal claims and consider alternative approaches to better approximate causal effects. To strengthen the methodological rigor, the authors could discuss potential strategies to mitigate these limitations. Explicitly addressing these aspects would enhance the validity of the findings and provide a more nuanced interpretation of the results.*

   Response: Thanks for this insightful advice.

   Scholarships are usually awarded before or at the start of each academic year based on applicants' qualifications, merit or need. Dropout, if it happens, can occur in the middle or at the end of the

academic term - but always after scholarship being awarded. Institutions generally don't wait to see how a student is doing before awarding scholarships. Covariates used in our propensity score model, e.g., gender, age at enrollment, can be considered either time-invariant or recorded prior to dropout. Therefore, the temporal sequencing of scholarship allocation is not a concern when discussing causality. In this study, a randomized controlled trial (RCT) is not feasible or inappropriate. For example, if a scholarship is aimed to be provided to students from low-income families or minority groups, then randomly issuing or denying the scholarship to qualified students would raise ethical concerns. How can a student be denied the scholarship simply because they are randomly put in a control group? Therefore, we find it necessary to employ a quasi-experimental approach to avoid ethical issues of random assignment, such as propensity score matching, to mimic the effects of RCT and to explore the influence of scholarship on dropout risks. However, we realize the limitations in propensity score matching and some potential alternatives to mitigate these limitations. When using propensity score matching, if there are some factors we cannot observe - like family pressure, mental health, etc, which may affect both scholarship receipt and dropout, then the validity of causal inference will be weakened. Regression discontinuity design (RDD) Guido et al. [7] doesn't have a strong reliance on observed covariates, and can be a better choice to approximate causal inference in RCT. For example, suppose that a scholarship's eligibility is income based, and we know the threshold income value such that everyone below gets the scholarship and otherwise not. Then, the characteristics of students just below and above the cutoff income value are likely very similar. RDD can mimic a RCT well, and estimate the causal effect of the scholarship by comparing the two groups without the concern of unobservable covariates. In this case, RDD compares the two groups more like a RCT than PSM without worrying whether all important covariates are measured. A case study can be found in Susan [8]. Such methods are worth exploration in future research with more informative data sets.

We write two new paragraphs in Section 3.2.5. to address this issue:

"In this study, a randomized controlled trial (RCT) is not feasible or inappropriate. For example, if a scholarship is aimed to be provided to students from low-income families or minority groups, then randomly issuing or denying the scholarship to qualified students would raise ethical concerns. Therefore, we find it necessary to employ a quasi-experimental approach to avoid ethical issues of random assignment, such as propensity score matching, to mimic the effects of RCT and to explore the influence of scholarship on dropout risks."

"To choose among the options, we assess covariate balance before and after matching using standardized mean differences (SMDs) and variance ratios. The matching method that results in the best balance is the "subclass" method. As shown in Figure 1, matching substantially reduced imbalance: after matching, nearly all covariates had absolute SMDs below 0.1, and variance ratios were within an acceptable range (most between 0.8 and 1.25). These diagnostics indicate that our matching procedure achieved strong balance between treated and control groups, improving the credibility of the causal estimates. Details of theoretical support of these diagnostic tools can be found in [9]. However, we want to caution that when using propensity score matching, unobserved confounders, such as family expectations or mental health, may bias causal estimates if they influence both scholarship receipt and dropout risk. Regression Discontinuity Design (RDD) proposed in [7] offers a viable alternative, as it relies on a known cutoff for treatment assignment and assumes local similarity around the threshold. For example, if a scholarship is awarded based on income, RDD can estimate the causal effect by comparing students just below and above the income cutoff. This method avoids reliance on full covariate adjustment and may better approximate a randomized experiment. A relevant case study discussing the causal effect of financial aid on university attrition can be found in [8]. Future research could explore such designs in more detail using richer datasets. "

5. *The discussion section requires greater depth and a more thorough interpretation of the results. While*

the main findings are presented, there is a lack of connection with existing literature, which limits the contextualization of the study's contributions. The manuscript would benefit from a more detailed comparison with previous research on university dropout rates and the impact of socioeconomic and academic factors. Additionally, there is insufficient evidence provided to frame the findings within broader academic discussions, making it difficult to assess their significance beyond the analyzed dataset. Strengthening these aspects by incorporating relevant references and discussing how the results align or diverge from prior studies would enhance the clarity and impact of the discussion.

Response: We write a new Discussion Section as follows. According to the editor's advice, we switch the order of the Discussion Section and the Conclusion Section.

"This study contributes to the literature on student dropout by integrating machine learning models with quasi-experimental analysis to evaluate both associative patterns and potential causal effects, particularly concerning financial aid. The results consistently identified financial hardship, academic disengagement, and student background characteristics as key predictors of dropout. The importance of financial vulnerability was strongly supported by both the GAM model and XGBoost feature importance. Being in debt, having unpaid tuition fees, and lacking a scholarship emerged as the most predictive financial variables—aligning closely with recent findings that emphasize financial instability as a major driver of student dropout [1, 3, 8]. Our analysis using propensity score matching also showed that receiving a scholarship significantly reduces the likelihood of dropout, echoing prior research that highlights the retention benefits of financial support [2,8]. These results reinforce ongoing calls for institutions to prioritize financial aid accessibility, scholarship expansion, and debt management programs as central to student success strategies. Academic engagement was another critical dimension of dropout risk. Students failing to complete second-semester courses were substantially more likely to leave their studies – aligning with findings from [10], which emphasized that predictive models based on first-year performance data can help identify high-risk students early in their academic journey. From a policy perspective, the implications for universities are clear: tracking academic and financial indicators in real time can allow institutions to identify students at risk and intervene before dropout occurs. For example, early alerts based on tuition payment status, course completion rates, or accumulated debt could initiate timely outreach through advising, tutoring, or emergency financial aid. This aligns with [2], who advocate for data-informed strategies that address both academic and socio-economic vulnerabilities.

This study has several limitations. First, the dataset used is five years old, and while it provides a robust historical overview, recent changes in higher education, such as remote learning or post-pandemic effects, may affect the generalizability of our findings. Second, the data were collected from a single public institution in Portugal. Institutional characteristics such as funding models, admissions policies, and student demographics differ widely across universities and countries. Thus, caution is warranted when generalizing these results to other contexts, especially outside of Portugal or within private institutions. Third, while propensity score matching adjusts for observed confounders, it cannot eliminate bias from unmeasured factors such as motivation, mental health, family pressure, or institutional support systems. These unobserved variables may influence both scholarship receipt and dropout, limiting the strength of causal inference. Finally, although the GAM model allowed for interpretability, it does not capture all possible interactions among important predictors.

In the future, improvements could be made by integrating data from multiple institutions or applying alternative causal inference techniques such as regression discontinuity design (RDD). RDD may be especially useful in settings where scholarship eligibility is based on a clear income or academic threshold, offering stronger quasi-experimental validity, as shown in [8]. Additionally, some potentially relevant variables, such as mental health, social integration, or employment status [2], were not available in our dataset. Integrating such information on student experiences could complement and

enrich model-driven insights. When working with extremely large datasets, deep learning models, such as neural networks, may outperform GAM or XGBoost in predictive accuracy due to their ability to capture complex, nonlinear interactions among variables. Future researchers might also apply emerging techniques from explainable AI (XAI), such as SHapley Additive exPlanations [11], to help improve interpretability of deep learning models. This could allow institutions to benefit from both the accuracy of neural networks and the transparency needed for actionable insights."

References

[1] Aina Carmen, Baici Eliana, Casalone Giorgia, Pastore Francesco The determinants of university dropout: a review of the socio-economic literature. Socio-Economic Planning Sciences. 2022;79. doi:10.1016/j.seps.2021.101102.

[2] Lorenzo-Quiles, Oswaldo, Galdón-López, Samuel, Lendínez-Turón, Ana Factors contributing to university dropout: a review. Frontiers in Education. 2023;8. doi:10.3389/feduc.2023.1159864.

[3] Raghul Gandhi Venkatesan, Dhivya Karmegam, Bagavandas Mappillairaju Exploring statistical approaches for predicting student dropout in education: a systematic review and meta-analysis. Journal of Computational Social Science. 2024;7:171-196. doi:10.1007/s42001-023-00231-w.

[4] Robert Tibshirani. Regression Shrinkage and Selection via the Lasso.1996;58(1);267–288. Journal of the Royal Statistical Society, Series B. doi:10.1111/j.2517-6161.1996.tb02080.x.

[5] Hastie T, Tibshirani R, Friedman J. The Elements of Statistical Learning: Data mining, Inference and Prediction. 2nd ed. New York, NY, USA: Springer; 2009.

[6] Chen T, Guestrin C. XGboost: a scalable tree boosting system. In: Proceedings of the 22nd ACM SIGKDD International Conference on Knowledge Discovery and Data Mining; 2016. p. 785–794. doi:10.1145/2939672.2939785.

[7] Guido W Imbens, Thomas Lemieux Regression discontinuity designs: a guide to practice. Journal of Econometrics. 2008;142(2):615-635. doi:10.1016/j.jeconom.2007.05.001.

[8] Susan M Dynarski Does aid matter? Measuring the effect of student aid on college attendance and completion. American Economic Review. 2003;93(1):279–288. doi:10.1257/000282803321455287.

[9] Peter C Austin An introduction to propensity score methods for reducing the effects of confounding in observational studies. Multivariate Behavioral Research. 2011;46(3):399–424. doi:10.1080/00273171.2011.568786.

[10] Thao-Trang Huynh-Cam, Long-Sheng Chen, Tzu-Chuen Lu. Early prediction models and crucial factor extraction for first-year undergraduate student dropouts.2024. Vol. ahead-of-print No. ahead-of-print. Journal of Applied Research in Higher Education. doi:10.1108.JARHE-10-2023-0461.

[11] Scott Lundberg, Su-In Lee A unified approach to interpreting model predictions. doi:10.48550/arXiv.1705.07874.
