# Supplementary material for: Statistical and machine learning models for predicting university dropout and scholarship impact
Source: PLoS One. 2025 Jun 25;20(6):e0325047. doi: 10.1371/journal.pone.0325047 (PMC12193850; doi:10.1371/journal.pone.0325047)
Supplement: Attachment — Submitted filename: responses_R3.pdf [file pone.0325047.s008.pdf]

Responses to Editor's Comments

We sincerely thank the Editor for their valuable suggestions and comments, which have significantly contributed to improving the rigor and overall quality of our manuscript.

Editor's Comments

1. *My first suggestion is to change the title. The current title does not reflect the complexity of the paper; it is very descriptive and more suited to an education-focused journal. The authors should consider alternative titles that incorporate both the models used and the main variable, which is being a scholarship holder. As a suggestion: "Statistical and Machine Learning Models for Predicting University Dropout and Scholarship Impact"*

   Response: Thanks. We change the title according to this advice.

2. *Introduction (first paragraph). It is also important to highlight that incomplete information about university degrees at the time of high school completion is often an additional factor. This lack of clarity affects students' decision-making, frequently leading to suboptimal academic choices. Furthermore, within a higher education production model, student dropouts represent a significant inefficiency that undermines the overall efficiency of the higher education system. (Add reference / Fig. 1) https://doi.org/10.1007/s10671-019-09254-5*

   Response: We add the following to the end of the first paragraph in the Introduction section: "In addition to academic and financial challenges, students may also face structural barriers that contribute to dropout risk. For example, incomplete information about university degrees at the time of high school graduation can affect students' decision-making and lead to suboptimal academic decisions. Such mismatches may set the stage for disengagement and eventual dropout. From a broader perspective, student attrition also represents an inefficiency within the higher education system, reducing the effectiveness of institutional investment and planning [1]."

3. *In section 3.2.3, the phrase "Because we are interested in predicting whether a student dropped out or graduated from their course" could indeed be rewritten for greater clarity for an international audience. A more precise alternative might be: "Since we aim to predict whether a student dropped out or successfully graduated from college."*

   Response: We change this phrase according to this advice.

4. *There are 25 final variables in the analysis, but the information presented in S1 (Table 4) is confusing for an international audience. Specifically, it should be clarified how many credits the courses in a semester have. The median value of 6 does not provide clarity. What is the grading system? A median score of 12.33 is not informative. Why is the inflation rate used? The data should be individual, corresponding to each student. When using group-level data, estimation issues arise, which can be addressed through multilevel regression. Overall, the table should explain what each variable measures.*

   Response: We appreciate the editor's comment regarding the role of inflation rate in the analysis. We used "inflation rate" for propensity score matching. It should not be used to build the predictive models because it is not an individual-level variable. We re-do the analysis without including "inflation rate" as a predictor. The evaluation metrics show only negligible changes (differences limited to the second or third decimal place), and the overall model rankings and variable importance remain unchanged. These results are included in Table 3 in the revised manuscript, with updates marked in blue.

At the end of Section 3.2.4, we change "After the preprocessing step, we finally end up with a data set with 3,400 observations and 25 variables for modeling." to be "After the preprocessing step, we finally end up with a data set with 3,400 observations and 25 variables for propensity score matching or modeling." We further add this paragraph to clarify the role of "inflation rate" and explain the variables with greater details: "5) national-level variable: with the exception of the inflation rate, all variables used in our analysis are measured at the individual level. The inflation rate is used solely as a macro-economic covariate in the propensity score matching procedure and is not included in the models predicting dropout." Regarding the suggestion to use multilevel modeling, we respectfully note that our data do not contain hierarchical group structures such as schools or regions. The inflation rate, being a national-level variable, does not define natural groupings and thus does not provide a meaningful multilevel structure. As all other variables are measured at the individual level, we consider multilevel regression to be not applicable in this case.

We have revised the S1 Table to make it clearer for an international audience. Specifically, we now provide information on the grading and credit system used in Portuguese higher education. Additionally, we add the following paragraph at the end of Section 3.2.4 to guide the reader: "Portuguese universities grade individual courses (also called curricular units) using a numeric scale from 0 to 20. The admission and previous qualification grades are on a 0 to 200 scale, consistent with the Portuguese higher education admission system. Several variables describe a student's academic activity in a given semester: the number of enrolled units reflects courses registered for; evaluated units indicate those in which at least one assessment was taken; approved units represent courses passed. Credited units may stem from prior recognition or transfer. The grade variable summarizes average performance across evaluated courses. These indicators provide complementary perspectives on academic engagement and success."

5. *In comment 5.3.1, "First, a scholarship can be a source of motivation." it should also be considered that students who do not meet the required academic performance lose their scholarship. In some cases, they may even be required to return the funds if their academic performance is extremely low or if they drop out.*

   Response: We add the following in Section 5.3.1: "Besides, many programs require recipients to maintain a minimum level of academic performance. Failure to meet these standards may result in the loss of ongoing scholarship eligibility. In some cases, when students' academic performance is extremely low or if they drop out, they may be required to repay funds."

References

[1] Salas-Velasco, Manuel The technical efficiency performance of the higher education systems based on data envelopment analysis with an illustration for the Spanish case. Educational Research for Policy and Practice. 2020; 19(2);159–180. doi:10.1007/s10671-019-09254-5.
